# SARS-CoV-2 spreads through cell-to-cell transmission

**DOI:** 10.1073/pnas.2111400119

**Published:** 2021-12-22

**Authors:** Cong Zeng, John P. Evans, Tiffany King, Yi-Min Zheng, Eugene M. Oltz, Sean P. J. Whelan, Linda J. Saif, Mark E. Peeples, Shan-Lu Liu

**Affiliations:** ^a^Center for Retrovirus Research, The Ohio State University, Columbus, OH 43210;; ^b^Department of Veterinary Biosciences, The Ohio State University, Columbus, OH 43210;; ^c^Molecular, Cellular, and Developmental Biology Program, The Ohio State University, Columbus, OH 43210;; ^d^Center for Vaccines and Immunity, Abigail Wexner Research Institute, Nationwide Children’s Hospital, Columbus, OH 43205;; ^e^Department of Pediatrics, The Ohio State University College of Medicine, Columbus, OH 43205;; ^f^Department of Microbial Infection and Immunity, The Ohio State University College of Medicine, Columbus, OH 43210;; ^g^Department of Molecular Microbiology, Washington University School of Medicine, St. Louis, MO 63110;; ^h^Center for Food Animal Health, Animal Sciences Department, Ohio Agricultural Research and Development Center, College of Food, Agricultural, and Environmental Sciences, The Ohio State University, Wooster, OH 44691;; ^i^Veterinary Preventive Medicine Department, College of Veterinary Medicine, The Ohio State University, Wooster, OH 44691;; ^j^Viruses and Emerging Pathogens Program, Infectious Diseases Institute, The Ohio State University, Columbus, OH 43210

**Keywords:** SARS-CoV-2, cell-to-cell transmission, cell–cell fusion, neutralization, variants of concern

## Abstract

Severe acute respiratory syndrome coronavirus 2 (SARS-CoV-2) is a highly transmissible coronavirus responsible for the global COVID-19 pandemic. Herein, we provide evidence that SARS-CoV-2 spreads through cell–cell contact in cultures, mediated by the spike glycoprotein. SARS-CoV-2 spike is more efficient in facilitating cell-to-cell transmission than is SARS-CoV spike, which reflects, in part, their differential cell–cell fusion activity. Interestingly, treatment of cocultured cells with endosomal entry inhibitors impairs cell-to-cell transmission, implicating endosomal membrane fusion as an underlying mechanism. Compared with cell-free infection, cell-to-cell transmission of SARS-CoV-2 is refractory to inhibition by neutralizing antibody or convalescent sera of COVID-19 patients. While angiotensin-converting enzyme 2 enhances cell-to-cell transmission, we find that it is not absolutely required. Notably, despite differences in cell-free infectivity, the authentic variants of concern (VOCs) B.1.1.7 (alpha) and B.1.351 (beta) have similar cell-to-cell transmission capability. Moreover, B.1.351 is more resistant to neutralization by vaccinee sera in cell-free infection, whereas B.1.1.7 is more resistant to inhibition by vaccinee sera in cell-to-cell transmission. Overall, our study reveals critical features of SARS-CoV-2 spike-mediated cell-to-cell transmission, with important implications for a better understanding of SARS-CoV-2 spread and pathogenesis.

## Significance

It is currently unknown if SARS-CoV-2 can spread through cell–cell contacts, and if so, the underlying mechanisms and implications. In this work, we show, by using lentiviral pseudotyped virus, that the spike protein of SARS-CoV-2 mediates the viral cell-to-cell transmission, with an efficiency higher than that of SARS-CoV. We also find that cell–cell fusion contributes to cell-to-cell transmission, yet ACE2 is not absolutely required. While the authentic variants of concern (VOCs) B.1.1.7 (alpha) and B.1.351 (beta) differ in cell-free infectivity from wild type and from each other, these VOCs have similar cell-to-cell transmission capability and exhibit differential sensitivity to neutralization by vaccinee sera. Results from our study will contribute to a better understanding of SARS-CoV-2 spread and pathogenesis.

SARS-CoV-2 is a novel beta-coronavirus that is closely related to two other highly pathogenic human coronaviruses, SARS-CoV and MERS-CoV (1). The spike (S) proteins of SARS-CoV-2 and SARS-CoV mediate entry into target cells, and both use angiotensin-converting enzyme 2 (ACE2) as the primary receptor (2–6). The spike protein of SARS-CoV-2 is also responsible for induction of neutralizing antibodies, thus playing a critical role in host immunity to viral infection (7–10).

Similar to HIV and other class I viral fusion proteins, SARS-CoV-2 spike is synthesized as a precursor that is subsequently cleaved and highly glycosylated; these properties are critical for regulating viral fusion activation, native spike structure, and evasion of host immunity (11–15). However, distinct from SARS-CoV, yet similar to MERS-CoV, the spike protein of SARS-CoV-2 is cleaved by furin into S1 and S2 subunits during the maturation process in producer cells (6, 16, 17). S1 is responsible for binding to the ACE2 receptor, whereas S2 mediates viral membrane fusion (18, 19). SARS-CoV-2 spike can also be cleaved by additional host proteases, including transmembrane serine protease 2 (TMPRSS2) on the plasma membrane and several cathepsins in the endosome, which facilitate viral membrane fusion and entry into host cells (20–22).

Enveloped viruses spread in cultured cells and tissues via two routes: by cell-free particles and through cell–cell contact (23–26). The latter mode of viral transmission normally involves tight cell–cell contacts, sometimes forming virological synapses, where local viral particle density increases (27), resulting in efficient transfer of virus to neighboring cells (24). Additionally, cell-to-cell transmission has the ability to evade antibody neutralization, accounting for efficient virus spread and pathogenesis, as has been shown for HIV and hepatitis C virus (HCV) (28–32). Low levels of neutralizing antibodies, as well as a deficiency in type I IFNs, have been reported for SARS-CoV-2 (18, 33–37) and may have contributed to the COVID-19 pandemic and disease progression (38–43).

In this work, we evaluated cell-to-cell transmission of SARS-CoV-2 in the context of cell-free infection and in comparison with SARS-CoV. Results from this in vitro study reveal the heretofore unrecognized role of cell-to-cell transmission that potentially impacts SARS-CoV-2 spread, pathogenesis, and shielding from antibodies in vivo.

## Results

### The Spike Protein of SARS-CoV-2 Efficiently Mediates Cell-to-Cell Transmission of Lentiviral Pseudotypes.

**Fig. 1.**
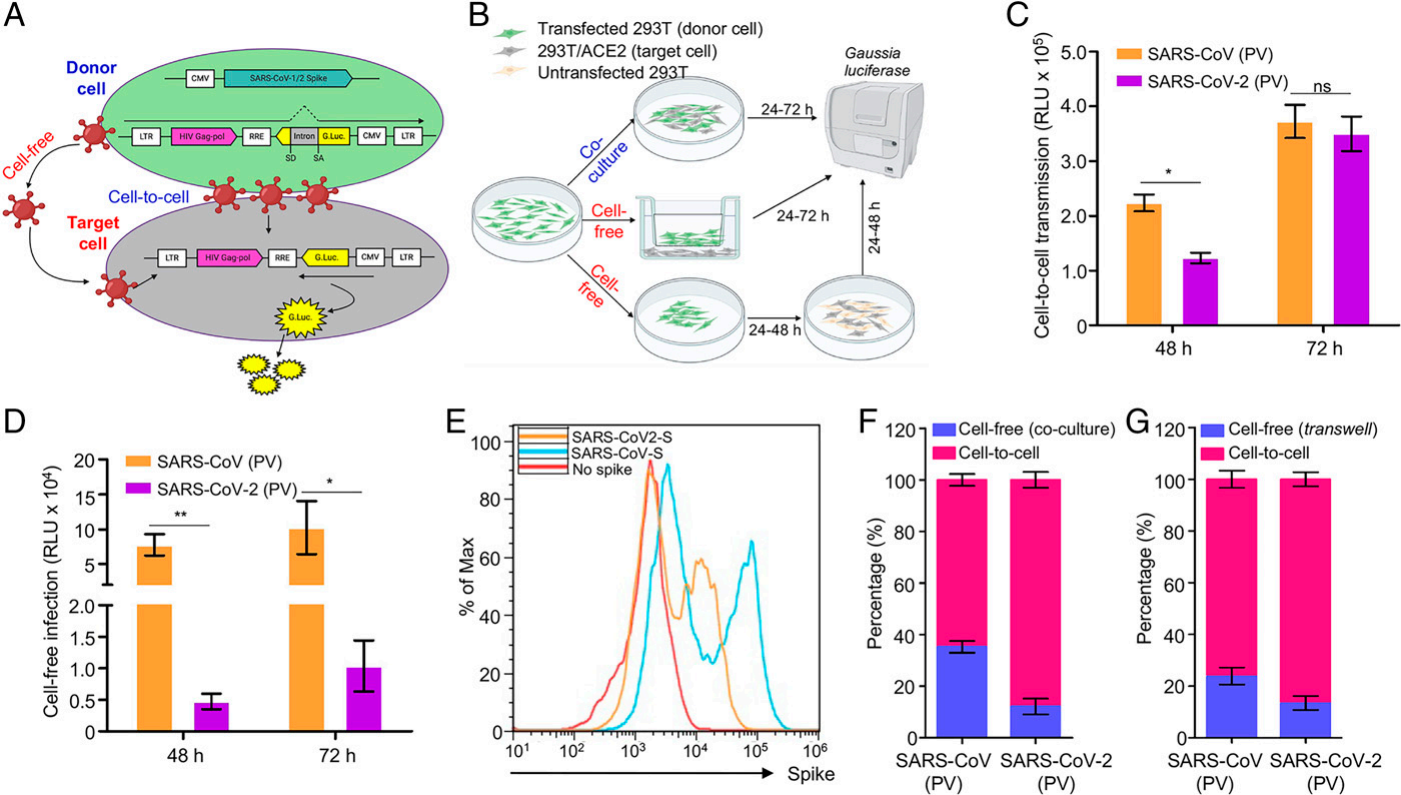
The spike protein of SARS-CoV-2 and SARS-CoV mediates cell-to-cell transmission of HIV-1 lentiviral pseudotypes. (*A* and *B*) Schematic representations of cell-to-cell and cell-free infection assays (see details in *Materials and Methods*). Briefly, the inGluc-based lentiviral pseudotypes bearing spike were produced in 293T cells, which were cocultured with the target cells (293T/ACE2) for cell-to-cell transmission; the Gluc activity of cocultured cells was measured over time (*A*). Cell-free infection was performed by harvesting virus from the same number of producer cells, followed by infecting 293T/ACE2 target cells in the presence of the same number of untransfected 293T cells; alternatively, cell-free infection was carried out in Transwell plates, from which Gluc activity was measured (*B*). (*C*) Comparison of cell-to-cell transmission mediated by SARS-CoV-2 or SARS-CoV spike. Results shown were from six independent experiments, with cell-free infection measured at 48 and 72 h after coculture; the portion of cell-free infection was excluded (*n* = 6). (*D*) Comparison of cell-free infection mediated by SARS-CoV-2 or SARS-CoV spike. Results were from six independent experiments (*n* = 6). (*E*) The expression level of spike proteins on the plasma membrane of donor cells was measured by flow cytometry using a polycolonal antibody T62, which detects both SARS-CoV-2 and SARS-CoV. (*F* and *G*) The calculated ratios between cell-to-cell and cell-free infection mediated by SARS-CoV-2 or SARS-CoV-2 spike. Results from cell coculture are shown in *F* and from Transwell plates shown in *G* (*n* = ∼3 to 6). PV, pseudotyped virus. *P < 0.05, **P < 0.01 . ns, not significant.

The spike is the only viral transmembrane protein that directly mediates SARS-CoV-2 entry into host cells. We evaluated whether the spike protein of SARS-CoV-2 is critical for viral spread through cell–cell contact. In order to compare the efficiency of cell-to-cell vs. cell-free infection mediated by the spike proteins of SARS-CoV-2 and SARS-CoV, we took advantage of an intron-Gaussia luciferase (inGluc) HIV-1 lentiviral vector bearing the spike of interest. In this system, the cells producing the inGluc lentiviral virions bearing the spike protein cannot themselves express Gluc because the intron is only removed during splicing of the virion genome transcribed from the integrated genome and not during the production of Gluc mRNA. However, when that lentivirus pseudotype enters a target cell, that genome is reverse transcribed and integrated into a new cell, and the cytomegalovirus promotor drives transcription of the now intron-less Gluc transcript leading to Gluc protein production (44, 45). We measured Gluc activity as a readout to compare the cell-to-cell and cell-free infection efficiencies (Fig. 1 *A* and *B* and *Materials and Methods*). Because cell-contact–mediated infection comprises both cell-to-cell transmission and cell-free infection, we calculated the efficiency of cell-to-cell transmission by subtracting the portion of cell-free infection performed in parallel (*Materials and Methods*).

Despite ∼2-fold lower level of SARS-CoV-2 cell-to-cell transmission compared with SARS-CoV after 48 h of coculturing of spike-bearing inGluc lentiviral pseudotype producer cells and 293T cells stably expressing human ACE2 (293T/ACE2), we observed similar levels of cell-to-cell transmission between SARS-CoV-2 and SARS-CoV by 72 h, indicating a more efficient spread of SARS-CoV-2 (Fig. 1*C*). In contrast, the rate of cell-free infection of SARS-CoV was much higher than that of SARS-CoV-2, i.e., ∼10-fold, as measured at 48 and 72 h postinfection (Fig. 1*D*). Flow cytometric analysis of viral producer cells using a polyclonal antibody that recognizes the S1 of both SARS-CoV-2 and SARS-CoV spikes showed that the fluorescence signal of SARS-CoV spike was higher than that of SARS-CoV-2 (Fig. 1*E*), in agreement with our previous report (46). By averaging results from six independent experiments, we estimated that cell-to-cell transmission contributed to >90% of the total SARS-CoV-2 spread in the coculturing system, as compared with ∼60% for SARS-CoV performed in identical experimental settings (Fig. 1*F*). Parallel experiments were also performed by using a Transwell system, which showed ∼90% cell-to-cell vs. ∼10% cell-free infection for SARS-CoV-2 compared with ∼77% cell-to-cell vs. ∼23% cell-free for SARS-CoV (Fig. 1*G*). Collectively, these results revealed that the spike protein of SARS-CoV-2 mediates cell-to-cell transmission of lentiviral pseudotypes more efficiently than the spike protein of SARS-CoV. However, the SARS-CoV spike is more capable of mediating cell-free infection compared with SARS-CoV-2 in the lentiviral pseudotyping system.

### Recombinant Vesicular Stomatitis Virus (rVSV) Expressing SARS-CoV-2 Spike Spreads Faster than rVSV Bearing SARS-CoV Spike.

**Fig. 2.**
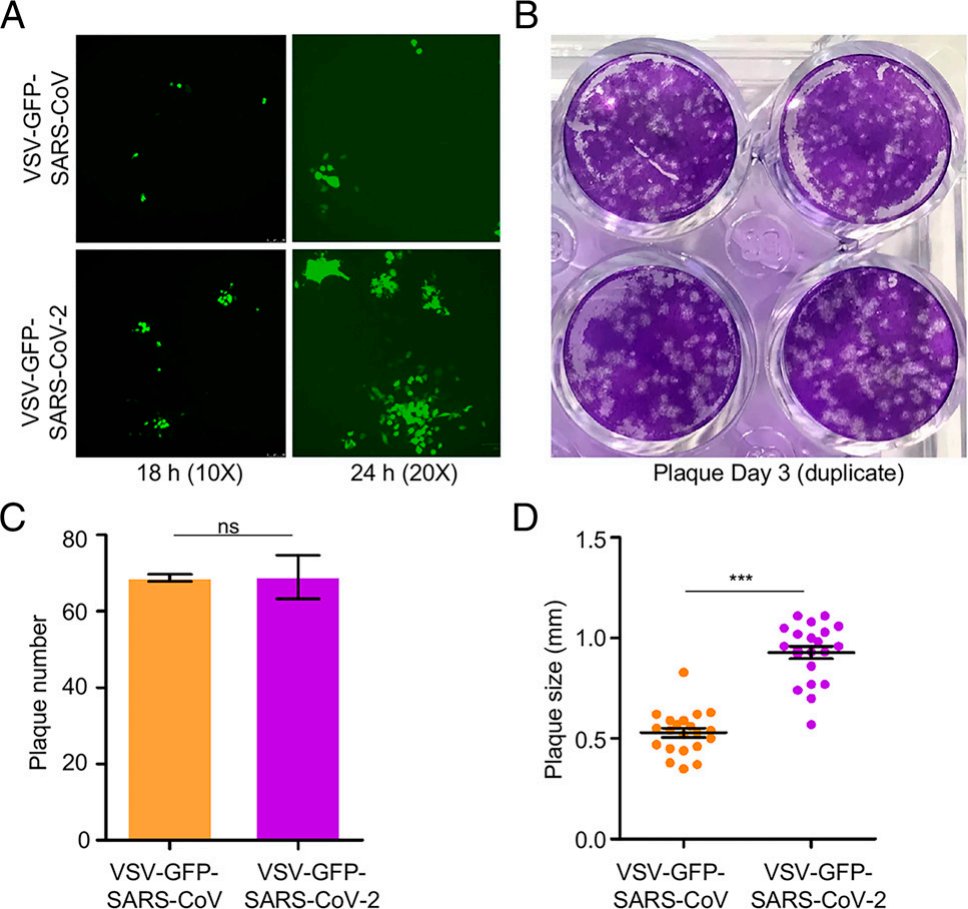
rVSV expressing SARS-CoV-2 spike spreads faster than does rVSV bearing SARS-CoV spike. Vero-E6 cells were infected with rVSV-GFP-SARS-CoV-2 or SARS-CoV (MOI = 0.01); 1 h postinfection, cells were washed with PBS and cultured in the presence of 1% methylcellulose. Photos were taken at 18 h and 24 h (*A*). After 72 h of infection, cells were fixed with 3.7% paraformaldehyde (PFA) and stained with crystal violet (*B*). The number and size of plaques are plotted in *C* and *D*, respectively. ***P < 0.001. ns, not significant.

We next compared the spreading infection of replication-competent rVSV expressing SARS-CoV-2 or SARS-CoV spike. This system has been previously used to study the cell-to-cell transmission of Ebolavirus (EBOV) mediated by the glycoprotein (GP) (30). Vero cells were inoculated with a relatively low multiplicity of infection (MOI) (0.01) of rVSV expressing GFP and SARS-CoV-2 spike in the place of VSV G protein (rVSV-GFP-SARS-CoV-2) or SARS-CoV spike (rVSV-GFP-SARS-CoV) (47). Cells were overlaid with 1% methylcellulose to block viral diffusion, and the number and size of GFP-positive plaques were stained and determined by fluorescence microscopy. Despite similar numbers of GFP-positive plaques between SARS-CoV-2 and SARS-CoV, which confirmed equivalent inoculations, the sizes for SARS-CoV-2 plaques were noticeably larger, as inspected at 18 and 24 h postinfection (Fig. 2 *A* and *B*). Quantitative analyses of data at 72 h showed that the size of SARS-CoV-2 plaques (diameter 0.93 ± 0.03 mm) was about two times greater than that of SARS-CoV (diameter 0.53 ± 0.02 mm), whereas the plaque numbers between SARS-CoV-2 and SARS-CoV were comparable (Fig. 2 *C* and *D*).

We next attempted to visualize cell-to-cell transmission of rVSV-GFP-SARS-CoV-2 by imaging fluorescent dye transfer in cocultured cells, either in the presence of methylcellulose or monoclonal antibody 2B04 against the SARS-CoV-2 spike. In this experiment, donor Vero cells were infected with rVSV-GFP-SARS-CoV-2 at different MOIs and subsequently cocultured with target Vero cells stably expressing mTomato (Vero-mTomato-Red). Efficient transmission was detected using fluorescence microscopy, as well as by flow cytometry at 6 h, with 23.9% double-positive cell populations (*SI Appendix*, Fig. S1 *A* and *B*). Treating cocultured cells with methylcellulose, which has been found to prevent cell-free infection by drastically reducing the diffusion of virions between cells (24), or 2B04 that potently inhibits cell-free infection (46), reduced the cell-to-cell transmission to 12.7% and 5.38%, respectively. Combining results from multiple independent experiments, we estimated that ∼50% of the total infection came from cell-to-cell transmission, which was still partially blocked by 2B04 (*SI Appendix*, Fig. S1*C*). Similar experiments performed in parallel for rVSV-GFP-SARS-CoV showed a stronger inhibition by methylcellulose (∼65%), suggesting a more efficient cell-free infection of rVSV-GFP-SARS-CoV compared with that of SARS-CoV-2. Importantly, 2B04 had no effect on cell-to-cell or cell-free infection of rVSV-GFP-SARS-CoV as would be expected since 2B04 does not cross-react with SARS-CoV (*SI Appendix*, Fig. S1 *D*–*F*) (46, 48). Altogether, these results demonstrated that, similar to lentiviral pseudotypes, the spike protein of SARS-CoV-2 more efficiently mediates the cell-to-cell transmission of rVSV-GFP than SARS-CoV.

### The Higher Cell–Cell Fusion Activity of SARS-CoV-2 Spike Contributes to Efficient Cell-to-Cell Transmission of the Pseudotyped Virus.

**Fig. 3.**
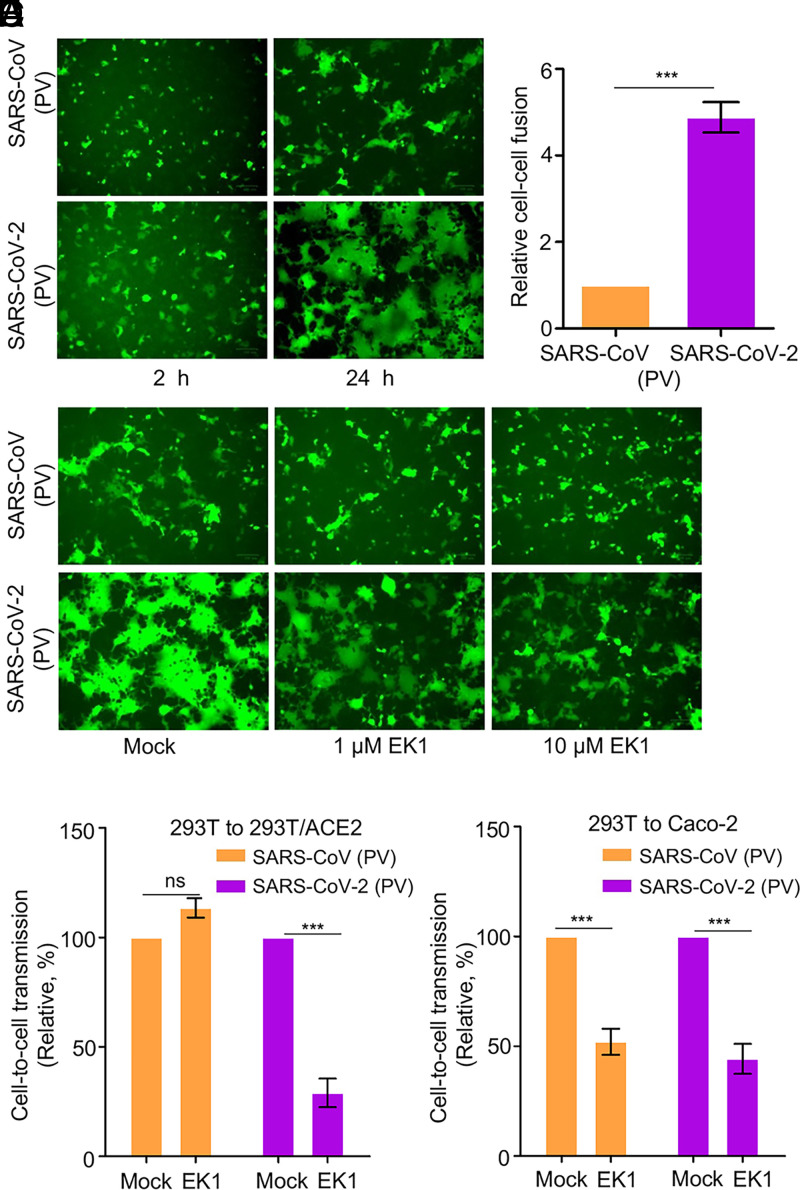
Cell–cell fusion mediated by SARS-CoV and SARS-CoV-2 spike contributes to cell-to-cell transmission. (*A*) Syncytia formation mediated by the spike of SARS-CoV-2 or SARS-CoV. The 293T donor cells were cotransfected with plasmids encoding SARS-CoV-2 or SARS-CoV spike, lentiviral NL4-3 inGluc vector, and eGFP. After 24 h posttransfection, the donor cells were cocultured with target 293T/ACE2 cells at a 1:1 ratio, with fusion monitored over time and photos taken after 2 h and 24 h, respectively. (*B*) Quantification of cell–cell fusion. The 293T cells were transfected with plasmids encoding tet-off or SARS-CoV or SARS-CoV-2 spike and cocultured with target 293FT-mCAT-Gluc cells, which were transfected with a plasmid expressing ACE2; Gluc activity was measured from the supernatant of cocultured cells at 24 h and 48 h, respectively. Relative fusion was plotted by setting the fusion activity of SARS-CoV as 1.0. (*C*–*E*) Fusion inhibitor EK1 inhibits cell–cell fusion of SARS-CoV-2 spike, in accordance with its effect on cell-to-cell transmission. Effect of EK1 on syncytia formation induced by SARS-CoV-2 spike (*C*); photos were taken at 24 h. Effects of EK1 on SARS-CoV-2 or SARS-CoV infection from 293T to 293T/ACE2 (*D*) or from 293T to Caco-2 (*E*). Transfected 293T donor cells were cocultured with 293T/ACE2 or Caco-2 cells in the presence or absence of 10 µM EK1, and Gluc activity was measured at 24 to 72 h after coculture. Results from three to six independent experiments were averaged and plotted as relative values by setting the mock control as 100% (*n* = ∼3 to 6). PV, pseudotyped virus. ***P < 0.001. ns, not significant.

We next explored whether cell–cell fusion by SARS-CoV-2 spike plays a role in cell-to-cell transmission. To this end, we cotransfected 293T cells with plasmids expressing the inGluc lentiviral vector, SARS-CoV-2 or SARS-CoV spike, and GFP. The transfected producer cells were cocultured with target 293T/ACE2 cells; syncytia formation and cell-to-cell transmission were measured over time. Following ∼2 h of coculturing, we observed small but apparent syncytia for SARS-CoV-2, yet with no syncytia formation for SARS-CoV (Fig. 3*A*). At 24 h following coculturing, more syncytia formation, with larger sizes, was observed in cells expressing SARS-CoV-2 spike, whereas fewer and smaller syncytia were seen for SARS-CoV (Fig. 3*A*). The difference between SARS-CoV-2 and SARS-CoV spike-induced cell–cell fusion was further evaluated by a more quantitative, Tet-off–based fusion assay, which showed an approximately fivefold higher fusion activity of SARS-CoV-2 compared with that of SARS-CoV (Fig. 3*B*).

We next treated cocultured cells with a pan-coronavirus fusion peptide inhibitor EK1 that has been shown to inhibit fusion of SARS-CoV-2, SARS-CoV, and other related CoVs (49, 50), and simultaneously measured its effect on cell–cell fusion and cell-to-cell transmission. Syncytia formation of SARS-CoV-2 was strongly inhibited by EK1 (Fig. 3*C*), in accordance with its effect on cell-to-cell transmission (Fig. 3*D*). Unexpectedly, although EK1 inhibited the ability of SARS-CoV spike to induce small syncytia, we did not find obvious inhibition of EK1 on SARS-CoV spike-mediated cell-to-cell transmission (Fig. 3 *C* and *D*). To investigate whether these results were cell-type dependent, we performed similar experiments using human intestine epithelial Caco-2 as target cells and found that EK1 indeed inhibited the cell-to-cell transmission of both SARS-CoV-2 and SARS-CoV (Fig. 3*E*). Overall, these results support the concept that the cell–cell fusion activity of SARS-CoV-2 and SARS-CoV spike contributes to cell-to-cell transmission, in a cell type–dependent manner, and that extensive syncytia formation could lead to cell death and therefore decreased transmission efficiency at the late stage of the processes.

### ACE2 Enhances but Is Not Required for Cell-to-Cell Transmission.

**Fig. 4.**
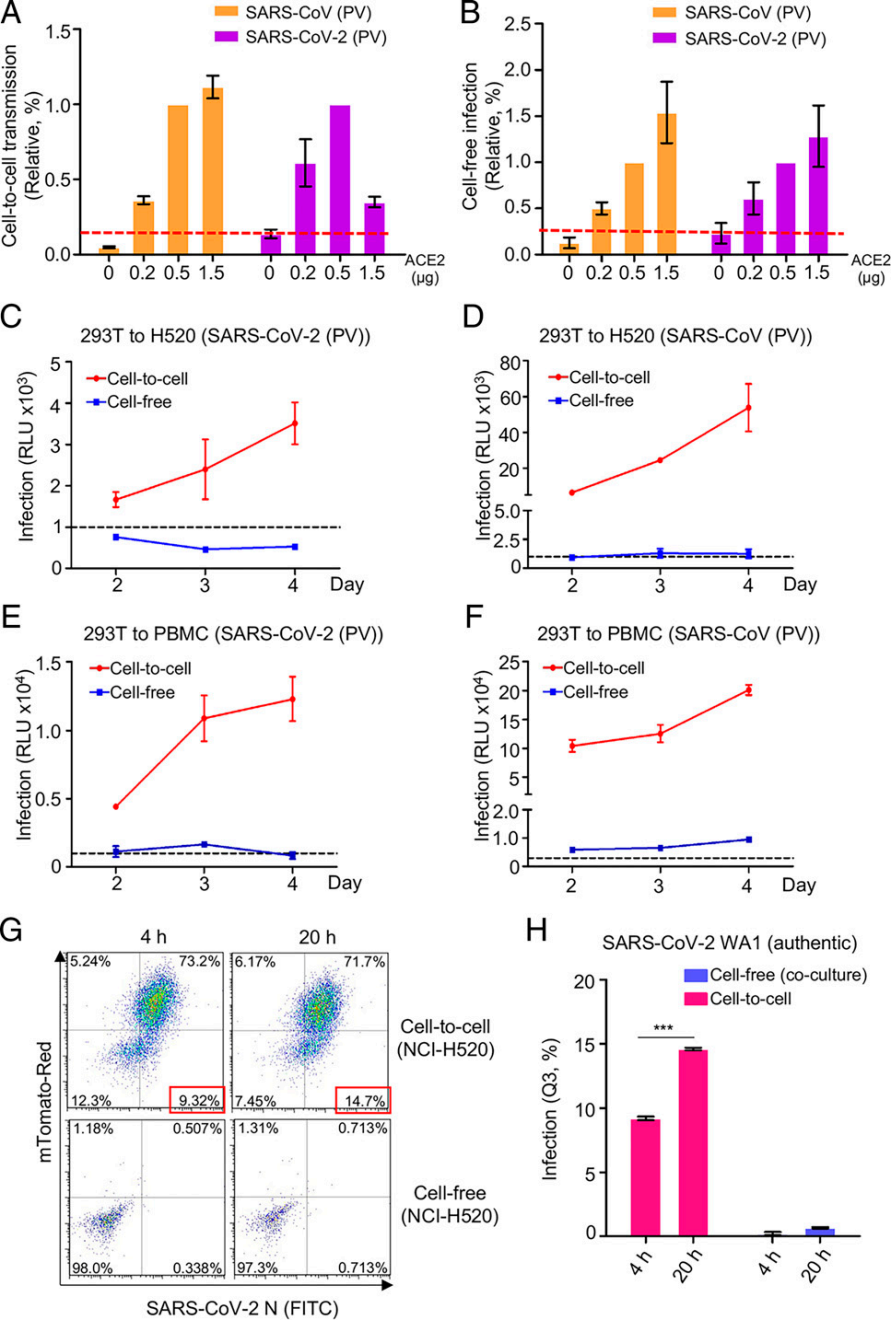
ACE2 enhances cell-to-cell transmission but is not absolutely required. (*A* and *B*) Cell-to-cell and cell-free infection were performed as described for Figs. 1 and 3 except that target cells were 293T transfected with different amounts of a plasmid encoding ACE2. Relative rates of cell-to-cell transmission and cell-free infection were calculated by setting the values of 0.5 µg ACE2 to 1.0 (*A* and *B*, *n* = 3). (*C*–*F*) Experiments were carried out as described for Figs. 1 and 3 except that target cells were H520 and human PBMCs (*n* = 3 for each). (*G*) Vero-ACE2-TMPRSS2-mTomato (Red) cells were infected with MOI = 0.01 of authentic SARS-CoV-2 WT (USA-WA1/2020) for 18 h, followed by coculturing of the donor NCI-H520 cells for another 4 h or 20 h. Cells were fixed and stained with an anti–SARS-CoV-2 N protein for flow cytometric analysis. The same number of NCI-H520 cells were infected with the WT SARS-CoV-2 harvested from the SARS-CoV-2–infected donor Vero-ACE2-TMPRSS2-mTomato cells for 4 h or 20 h for cell-free infection and analyzed by flow cytometry. (*H*) Results of Q3 quadrant analyses were plotted. ***P < 0.001. PV, pseudotyped virus.

ACE2 is the primary receptor of both SARS-CoV-2 and SARS-CoV, mediating viral entry into host cells. We next evaluated the role of ACE2 in cell-to-cell transmission as compared with cell-free infection. We observed increased cell-to-cell and cell-free infection when more plasmid encoding ACE2 was transfected into the target 293T cells, as would be expected (Fig. 4 *A* and *B*). Interestingly, with a relatively low dose of ACE2 (i.e., 0.2 μg), SARS-CoV-2 reached ∼70% of its maximal cell-to-cell transmission (at 0.5 μg ACE2). In contrast, SARS-CoV showed ∼30% maximal cell-to-cell transmission at 1.5 μg ACE2 (Fig. 4 *A* and *B*). Notably, when the highest dose of ACE2 (1.5 μg) was transfected into target cells, we consistently observed decreased cell-to-cell transmission of SARS-CoV-2 compared with a continually increasing trend for SARS-CoV (Fig. 4 *A* and *B*). This pattern of cell-to-cell transmission was different from that of cell-free infection, where both SARS-CoV-2 and SARS-CoV exhibited an increase, with similar kinetics, in a strictly ACE2 dose–dependent manner (Fig. 4 *A* and *B*). We confirmed ACE2 expression in target cells by flow cytometry and Western blotting (*SI Appendix*, Fig. S2 *A* and *B*). Consistent with increasing expression of ACE2 in target cells, we observed increasing sizes of syncytia formation for SARS-CoV-2, but cell–cell fusion by SARS-CoV was not evident (*SI Appendix*, Fig. S2*C*). Giant syncytia formation at 1.5 μg ACE2 resulted in cell death, which might have contributed to decreased cell-to-cell transmission for SARS-CoV-2 (*SI Appendix*, Fig. S2*C*). Overall, these results indicate that ACE2 enhances cell-to-cell transmission of both SARS-CoV-2 and SARS-CoV in the lentiviral pseudotyping system, yet the former requires less ACE2 for the process to occur.

We further explored whether cell-to-cell transmission of SARS-CoV-2 can occur in the absence of ACE2 expression in target cells. We first used NCI-H520, a human lung epithelial cell line that expresses an extremely low level of ACE2 (*SI Appendix*, Fig. S2*D*). Cell-to-cell transmission was detected at day 2, which continued to increase through day 4. In contrast, cell-free infection was not detected in NCI-H520 cells throughout the 3-d period (Fig. 4*C*). Cell-to-cell transmission was also observed for SARS-CoV in H520 cells, at a higher level than that of SARS-CoV-2; but again, similar to SARS-CoV-2, no/low cell-free infection was detectable (Fig. 4*D*). We next tested human peripheral blood mononuclear cells (PBMCs), which do not express ACE2 (*SI Appendix*, Fig. S2*D*), and observed apparent cell-to-cell transmission for both SARS-CoV and SARS-CoV-2, yet no/low cell-free infection was detected, the latter being consistent with recently published results (51) (Fig. 4 *E* and *F*). As a control, we carried out cell-to-cell transmission and cell-free infection in Calu-3, a human lung epithelial cell line that expresses a higher level of ACE2 (*SI Appendix*, Fig. S2*D*). A rapid increase in cell-to-cell transmission was observed for SARS-CoV-2 from day 2 through day 4, despite an overall level of infection for SARS-CoV that was higher than observed for SARS-CoV-2 (*SI Appendix*, Fig. S2 *E* and *F*). Together, these results demonstrated that cell-to-cell transmission of lentiviral pseudotypes bearing SARS-CoV-2 or SARS-CoV spike can occur in the absence of ACE2.

We next examined cell-to-cell infection of low-ACE2 H520 cells using authentic SARS-CoV-2 under biosafety level 3 (BSL3) conditions. We infected Vero-ACE2-TMPRSS2-mTomato cells with SARS-CoV-2 (USA-WA1/2020), which served as donor cells, and we cocultured them with H520 target cells for different periods of time. The cell-to-cell infection efficiency was determined by detecting the SARS-CoV-2 N protein in H520 cells using flow cytometry. In parallel, the cell-free infection of H520 cells was also analyzed. At 4 and 20 h following coculturing, we observed that 9.32 to 14.7% of H520 cells became positive for the SARS-CoV-2 N protein, whereas less than 1% of H520 cells were positive in cell-free infection (Fig. 4 *G* and *H* and *SI Appendix*, Fig. S2*G*). These results indicated that the authentic SARS-CoV-2 can infect H520 cells expressing a very low level of ACE2.

### Cell-to-Cell Transmission of SARS-CoV-2 Involves Endosomal Entry.

**Fig. 5.**
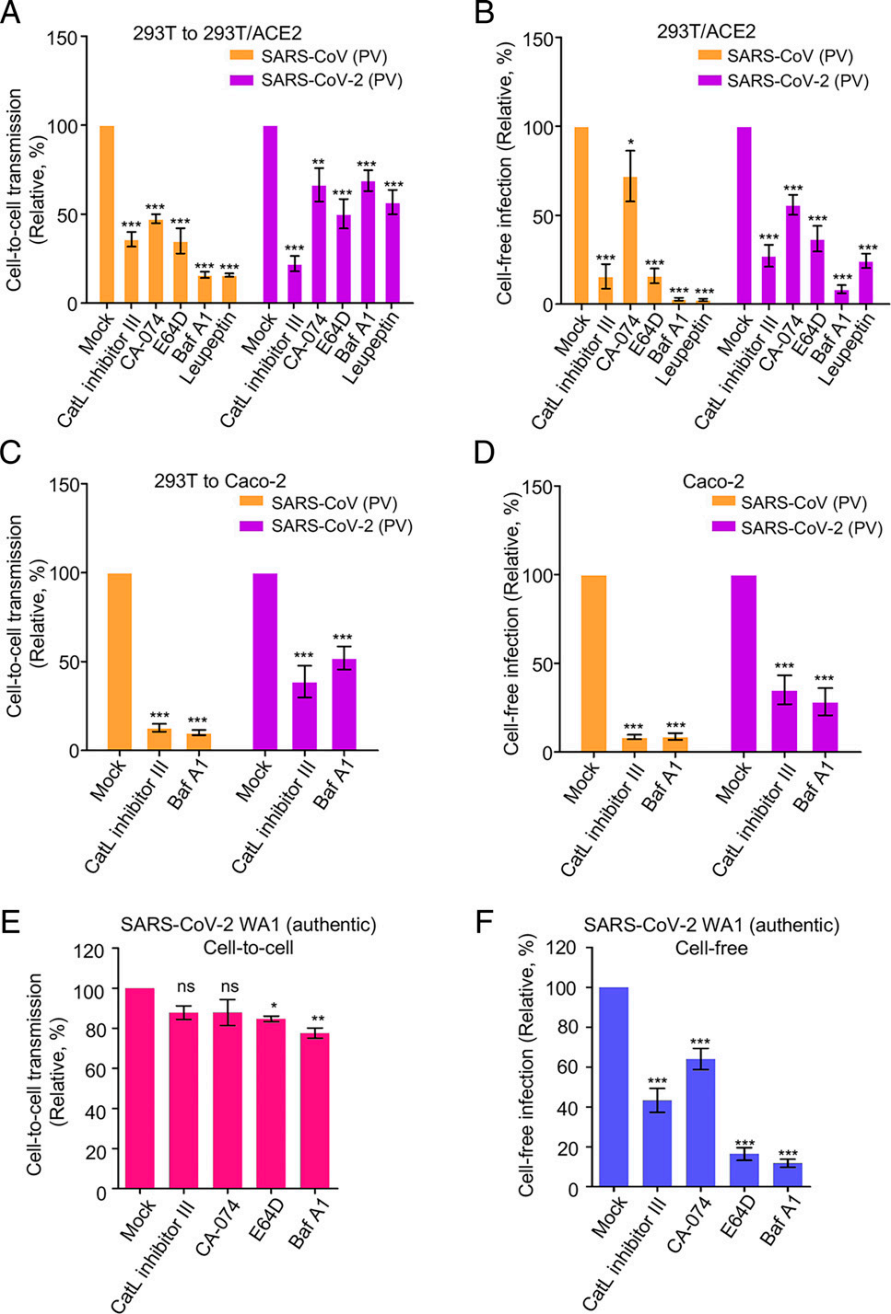
Endosomal entry pathway is involved in cell-to-cell transmission. Effect of endosomal entry inhibitors on cell-to-cell and cell-free infection of SARS-CoV-2 and SARS-CoV. Experiments were carried out as described in Fig. 1 *C* and *D*, except that indicated inhibitors were present during the infection period. The concentrations of inhibitors used were as follows: 1 µM or 5 µM Cat L inhibitor III, 1 µM or 5 µM CA-074, 10 µM or 30 µM E-64D, 25 nM or 50 nM BafA1, and 20 µM or 50 µM leupeptin. (*A* and *B*) Effect in 293T cells. (*C* and *D*) Effect in Caco-2 cells. In all experiments, Gluc activity was measured at 48 and 72 h after infection, and rates of relative infection were plotted by setting the values of mock infection without drugs to 100. Results were from approximately four to six independent experiments. (*E* and *F*) Effect of inhibitor treatments on cell-to-cell and cell-free infection of authentic SARS-CoV-2 (USA-WA1/2020). Note that Vero-ACE2-TMPRSS2-mTomato (Red) cells served as donor cells, and Vero-ACE2-TMPRSS2 cells served as target cells (*n* = 3). PV, pseudotyped virus. *P < 0.05, **P < 0.01, ***P < 0.001. ns, not significant.

SARS-CoV-2 uses different pathways for entry, either at the plasma membrane and/or in the endosomal compartment (20, 52–56). While our results indicated that entry via the plasma membrane is important for cell-to-cell transmission, we probed whether fusion in the endosomal compartment may also be involved. We applied in parallel a panel of endosomal inhibitors to the cell-to-cell and cell-free infection assays. We found that cathepsin L inhibitor III, cathepsin B inhibitor CA-074, E-64d (general cathepsin inhibitor), BafA1 (ATPase pump inhibitor), and leupeptin (general protease inhibitor), all significantly inhibited cell-to-cell transmission (Fig. 5*A*). Interestingly, the effect of these drugs on SARS-CoV-2 were generally less potent compared with SARS-CoV, with the exception of cathepsin L inhibitor III (Fig. 5*A*). Moreover, these drugs generally showed a stronger effect on cell-free infection, again especially for SARS-CoV (Fig. 5*B*). Of note, CA-074 had modest effects on both viruses (Fig. 5*B*), which was consistent with the notion that cathepsin B does not play a significant role in cleaving the spike protein of SARS-CoV and SARS-CoV-2, which is required for fusion (57, 58). We also applied these inhibitors to cell–cell fusion assays but found no effect on either SARS-CoV-2 or SARS-CoV, as would be expected (*SI Appendix*, Fig. S3). To assess possible cell type–dependent effects, we carried out experiments using Caco-2 target cells and found that cathepsin L inhibitor III and BafA1 robustly inhibited cell-to-cell transmission and cell-free infection of both viruses, in particular SARS-CoV (Fig. 5 *C* and *D*). Overall, these results support the notion that endosomal entry is involved in cell-to-cell transmission of SARS-CoV-2, and to a greater extent, SARS-CoV of lentiviral pseudotypes.

We further tested the effects of these inhibitors using authentic SARS-CoV-2. We infected Vero-ACE2-TMPRSS2-mTomato (Red) cells with SARS-CoV-2 (USA-WA1/2020), which were subsequently cocultured with either Vero-ACE2-TMPRSS2 or Calu-3 target cells in the presence of these inhibitors for 4 h. Flow cytometric analysis was used to detect virus-infected cells with an anti-N antibody of SARS-CoV-2. Similar to the results of lentiviral pseudotypes, much weaker effects were observed for these inhibitors on cell-to-cell spread as compared with cell-free infection (Fig. 5 *E* and *F* and *SI Appendix*, Fig. S4). Together, these results suggested that the endosomal entry pathway is involved in cell-to-cell transmission of authentic SARS-CoV-2, but it appears to play a less dominant role compared with cell-free infection.

### Cell-to-Cell Transmission of SARS-CoV-2 Is Refractory to Neutralizing Antibody and Convalescent Plasma.

**Fig. 6.**
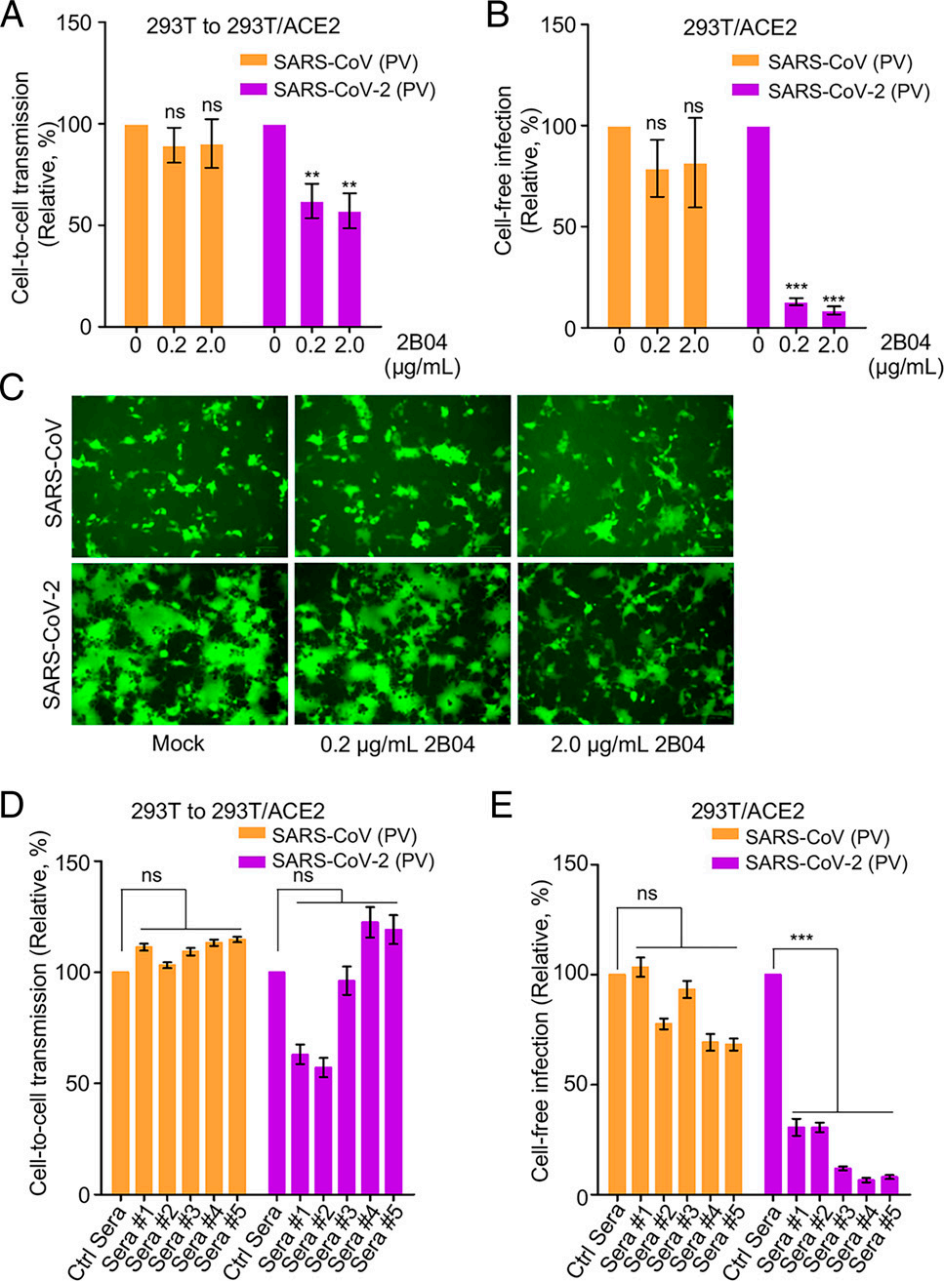
Cell-to-cell transmission of SARS-CoV-2 is refractory to inhibition by neutralizing antibody and COVID-19 convalescent plasma. (*A*–*C*) Effects of SARS-CoV-2 monoclonal antibody 2B04 on cell-to-cell transmission, cell-free infection, and cell–cell fusion mediated by SARS-CoV-2 or SARS-CoV-2 spike. The experiments were carried out as described in Fig. 1 *C* and *D*, except that 0.2 µg/mL or 2 µg/mL 2B04 were included during the infection period. Relative infections were plotted by setting the values of mock infection without 2B04 to100% for statistical analyses (*A* and *B*). The photos of syncytia formation were taken at 18 h after coculture and presented (*C*). (*D* and *E*) Effect of COVID-19 sera on cell-to-cell and cell-free infection of SARS-CoV-2 and SARS-CoV. Experiments were performed as described as above, except five diluted COVID-19 sera were included during the infection period. Effect on cell-to-cell (*D*) and cell-free (*E*) infection of SARS-CoV or SARS-CoV-2 were summarized and plotted by setting the values of mock infection control to 100% (*n* = ∼3 to 4). PV, pseudotyped virus. **P < 0.01, ***P < 0.001. ns, not significant.

One important feature of the virus cell-to-cell transmission is evasion of host immunity, particularly neutralizing antibody-mediated response. We therefore examined the sensitivity of SARS-CoV-2 spike-mediated cell-to-cell transmission to neutralization by a monoclonal antibody against the receptor-binding domain of the spike, 2B04 (48), as well as convalescent plasma derived from COVID-19 patients (46, 59). While 2B04 effectively inhibited cell-free infection of SARS-CoV-2 in 293T/ACE2 cells by more than 90%, its effect on cell-to-cell transmission between 293T and 293T/ACE2 was ∼50% (Fig. 6 *A* and *B*). As would be expected, 2B04 had no effect on SARS-CoV, regardless of cell-to-cell transmission or cell-free infection (Fig. 6 *A* and *B*). We also performed cell–cell fusion in the presence of different concentrations of 2B04, and we found that the fusion activity of the SARS-CoV-2 spike was inhibited in a dose-dependent manner (Fig. 6*C*). We then tested five serum samples of COVID-19 patients and observed that, although they potently inhibited the cell-free infection of SARS-CoV-2 (*P* < 0.001), they showed variable but no significant effect on cell-to-cell transmission of SARS-CoV-2; the effect of these sera on SARS-CoV infection, either cell-to-cell or cell-free, was minimal or modest (Fig. 6 *D* and *E*). Together, these results indicate that cell-to-cell transmission of SARS-CoV-2 lentiviral pseudotyped virus is mostly refractory to neutralization by neutralizing antibodies against spike relative to cell-free infection.

### Cell-to-Cell Transmission of Authentic SARS-CoV-2 Variants of Concern and Their Sensitivity to COVID-19 Vaccinee Sera.

The D614G mutation in SARS-CoV-2 spike, as well as emerging variants of concern (VOCs) containing D614G and other key spike mutations, have been reported to enhance viral infectivity, transmissibility, and resistance to COVID-19 vaccines (60–65). As such, we examined the cell-to-cell transmission capability of authentic SARS-CoV-2 wild type (WT) (USA-WA1/2020), D614G variant (B.1.5), and two VOCs B.1.1.7 (501Y.V1) and B.1.351 (South African, 501Y.V2), in the presence or absence of pooled sera from mRNA vaccines (three from Moderna and three from Pfizer). Donor Vero-ACE2 cells were first infected with WT SARS-CoV-2 (MOI = 0.2), D614G (MOI = 0.02), B.1.1.7 (MOI = 0.02), and B.1.351 (MOI = 0.02), respectively. Note that a 10-fold higher MOI was used for WT in order to achieve comparable rates of infection in donor cells between WT and VOCs, given that D614G-containing variants are known to significantly increase the viral infectivity (60, 66). Approximately 20 h postinfection, the culture media of donor cells was harvested, the whole volume of which was used to infect target Vero-mTomato-Red cells for 6 h in order to determine the viral infectivity. In parallel, the infected donor Vero-ACE2 cells were digested and cocultured with the same number of Vero-Tomato-Red cells as was used in the cell-free infectivity assay, also for 6 h, as a measurement of cell-to-cell transmission. To determine the sensitivity of cell-to-cell transmission vs. cell-free infection to neutralization by vaccinee sera, we pooled the serum samples of 6 mRNA vaccines, i.e., three from Moderna and three from Pfizer, and added them to the cultured medium. The efficiency of cell-to-cell transmission and cell-free infectivity was determined by measuring the percentage of SARS-CoV-2 nucleocapsid (N)-positive Vero-mTomato-Red cells using flow cytometry. Considering the potential impact of infected donor cells on cell-to-cell transmission, we normalized the rate of cell-to-cell transmission with the total rate of virus spread in both SARS-CoV-2–positive Vero-mTomato-Red cells as well as Vero-ACE2 cells over the entire infection period, i.e., from the initial infection of donor cells to the end of the coculture (∼26 h).

**Fig. 7.**
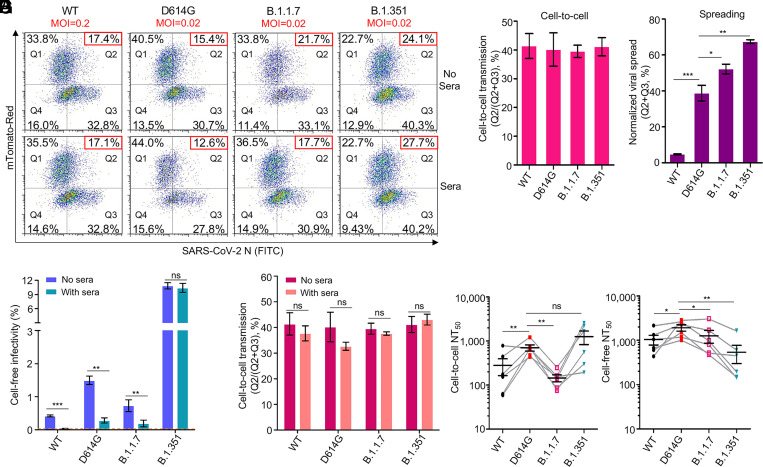
Cell-to-cell transmission of SARS-CoV-2 VOCs and sensitivity to neutralization by vaccinee sera. (*A*–*E*) The cell-to-cell transmission capability of authentic SARS-CoV-2 WT, D614G, B.1.1.7, and B.1.351 in the presence or absence of vaccinee sera. Donor Vero-ACE2 cells were infected with WT SARS-CoV-2 (MOI = 0.2), D614G (MOI = 0.02), B.1.1.7 (MOI = 0.02), and B.1.351 (MOI = 0.02) for 20 h, followed by coculturing with target Vero-mTomato (Red) cells in the presence or absence of pooled mRNA vaccinee sera (three from Moderna and three from Pfizer) for 6 h. Cells were fixed and stained with anti–SARS-CoV-2 N protein and analyzed by flow cytometry. Representative flow cytometric analyses of infected cells are shown in *A*, with the newly infected target Vero-mTomato (Red) cells (Q2) as indicative of cell-to-cell transmission. The relative cell-to-cell transmission efficiency was calculated by dividing the rate of Vero-mTomato-Red positive cells (Q2) by the rate of total infected donor and target cells (Q2+Q3) (*B*, *n* = 3). The MOI-normalized total viral spread in both donor and target cells (Q2+Q3) is shown in *C* (*n* = 3). The supernatant from the initial 20-h infection of donor cells was used to infect target Vero-mTomato-Red cells for 6 h as the measurement of cell-free viral infectivity, either in the presence or absence of the pooled vaccinee sera, and infected cells were analyzed by flow cytometry (*D*) (*n* = 3). The pooled vaccinee sera were also added to the cocultured Vero-ACE2 and Vero-mTomato-Red cells as described in *A* to determine their effect on cell-to-cell transmission (*E*). (*F* and *G*) The calculated NT_50_ values of vaccine sera against cell-to-cell transmission and cell-free infection of lentiviral pseudotypes bearing individual spike of VOCs. Experimental procedures were the same as described in Fig. 6 *D* and *E*, except that all comparisons were made relative to the D614G variant (*n* = 6). *P < 0.05, **P < 0.01, ***P < 0.001. ns, not significant.

Representative flow cytometric results and summary analyses are presented in Fig. 7 and *SI Appendix*, Fig. S5. Interestingly, even with a 10-fold higher MOI used for the WT infection of donor Vero-ACE2 cells relative to other variants, we observed comparable rates of cell-to-cell transmission between WT, D614G, B.1.1.7, and B.1.351 (Fig. 7 *A*, *Upper* and *B* and *SI Appendix*, Fig. S5 *A* and *B*). Note that the relative rate of cell-to-cell transmission shown in Fig. 7*B* was obtained by dividing the percentage of SARS-CoV-2–positive Vero-mTomato-Red cells (Q2 in Fig. 7 *A*, *Upper*) by the percentage of total SARS-CoV-2–positive cells (Q2 plus Q3 in Fig. 7 *A*, *Upper*). We noted that the rate of B.1.351 spreading infection in Vero-ACE2 and Vero-mTomato-Red cells (Q2 plus Q3 in Fig. 7 *A*, *Upper*) was the highest, followed by B.1.1.7 > D614G > WT (Fig. 7*C*). Consistent with the more efficient replication of B.1.351 in donor Vero-ACE2 cells over the entire 26-h infection period (Q3 in Fig. 7 *A*, *Upper*), we found a significantly higher cell-free infectivity for B.1.351 produced during the initial 20-h infection relative to WT, D614G, and B.1.1.7 (Fig. 7*D*, see “no sera”). Overall, these results revealed a strongly enhanced replication of B.1.351 relative to B.1.1.7, D614G, and WT, yet a comparable efficiency of cell-to-cell transmission between WT, D614G, and VOCs.

We also assessed the sensitivity of cell-to-cell transmission and cell-free infection to neutralization by Moderna and Pfizer vaccinee sera. With a relatively low dose of pooled sera being applied, we observed that the cell-to-cell transmission of WT, D614G, B.1.1.7, and B.1.351 was virtually resistant to neutralizing antibodies induced by these mRNA vaccines for all viruses, whereas the cell-free infection of WT, D614G, and B.1.1.7 was strongly inhibited, with B.1.351 being resistant (Fig. 7 *A*, *Lower* and *D* and *E* and *SI Appendix*, Fig. S5 *C* and *D*). By using HIV-inGluc pseudotyped viruses with serially diluted serum samples from Moderna and Pfizer vaccines, we were able to obtain and compare the 50% neutralizing titer (NT_50_) values of each virus in cell-to-cell transmission vs. cell-free infection. We found that, overall, mRNA vaccinee sera neutralized cell-to-cell transmission ∼3-fold less efficiently than cell-free infection, with the notable exception of B.1.351, which showed similar extents of inhibition for cell-to-cell and cell-free infections (Fig. 7 *F* and *G*). Intriguingly, we found that the cell-to-cell transmission of B.1.1.7 was more resistant to neutralization by vaccinee sera, with ∼4.9-fold lower NT_50_ than D614G (*P* < 0.01) and ∼8.7-fold lower than B.1.351 (*P* < 0.05) (Fig. 7 *F* and *G*). In contrast, the cell-free infection of B.1.351 was more resistant to neutralization than D614G and B.1.1.7, with 3.6-fold (*P* < 0.01) and ∼2.4-fold (*P* < 0.01) lower NT_50_, respectively (Fig. 7 *F* and *G*), which was consistent with recent studies (67, 68). In aggregate, these results confirmed that cell-to-cell transmission of both authentic and pseudotyped SARS-CoV-2 VOCs is more refractory to inhibition by neutralizing antibodies induced by mRNA vaccines as compared with cell-free infection, and more importantly, showed that the cell-to-cell transmission of B.1.1.7 and the cell-free infection of B.1.351, are most resistant to antibody neutralization. The differential sensitivity of B.1.1.7 and B.1.351 to neutralization by vaccinee sera in cell-to-cell transmission vs. cell-free infection likely has important implications for understanding the spread of these variants and their pathogenesis in patients (see *Discussion*).

## Discussion

Accumulating evidence indicates that viruses, including the highly pathogenic HIV, HCV, and EBOV, etc., can efficiently spread through cell-to-cell transmission (23, 29–31, 69–71). Importantly, cell-to-cell transmission is more efficient than cell-free infection (31), and roles for this mode of transmission have been demonstrated in vivo for HIV and other viruses (29, 31, 45, 70). Notably, many plant viruses are known to use cell-to-cell transmission to spread from epidermal cells and move sequentially into mesophyll, bundle sheath, and phloem parenchyma and companion cells (72, 73). For coronaviruses, very little is currently known about their mode of spread between cells or its efficiency compared with cell-free infection. This question is critical, given the robust replication of SARS-CoV-2 in human lung and other tissues, as well as the rapid spread of SARS-CoV-2, including some variants of concern, in the human population, leading to the global pandemic (38, 67, 68, 74, 75). In this work, we addressed this question using lentiviral pseudotypes, replication-competent rVSV expressing the spike of SARS-CoV-2 or SARS-CoV, as well as authentic SARS-CoV-2. We discovered that SARS-CoV-2 spike is more efficient in mediating cell-to-cell transmission than SARS-CoV spike, yet the spike of SARS-CoV is more capable of mediating cell-free infection. To our knowledge, this is a direct comparison of cell-to-cell transmission vs. cell-free infection between SARS-CoV-2 and SARS-CoV in cultured cells, and the results provide important insights into two distinct modes of infection and the host–viral factors that regulate these processes.

We provide evidence that the relatively robust cell-to-cell infection efficiency of SARS-CoV-2 is in part related to the higher cell–cell fusion capability of its spike protein compared with that of SARS-CoV (Fig. 3). Further evidence supporting a role of cell–cell fusion in transmission of SARS-CoV-2 came from the application of a membrane fusion inhibitor EK1, which significantly attenuated cell-to-cell transmission. Indeed, cell–cell fusion has been recognized as an important mechanism of cell-to-cell infection for a number of enveloped viruses, including herpesviruses, paramyxoviruses, and retroviruses (23). However, it must be emphasized that extensive cell–cell fusion by SARS-CoV-2 spike can lead to giant syncytia formation and cell death, which in turn reduces cell-to-cell transmission. While we were able to confirm the cell-to-cell infection of SARS-CoV-2 using the authentic WA-1 strain, syncytia formation was not evident in most cases, except when Vero-ACE2-TMPRRS2-mTomato and Vero-ACE2-TMPRRS2 cells were used as donor and target cells, respectively, because of the high level expressions of both ACE2 and TMPRSS2. However, syncytia per se shall not account for the transfer of SARS-CoV-2 N protein from donor cells to target cells, because cells were completely digested by trypsin and fused cells should have been excluded based on the single cell analysis using forward scatter area (FSC-A)/forward scatter width (FSC-W) gating. In this respect, it is worthwhile noting that authentic SARS-CoV-2 infection can induce giant syncytia in human lung epithelial H1299-ACE2 cells (76), although the size of syncytia induced by SARS-CoV-2 can be cell type–dependent (77). Overall, a fine control of the spike-induced cell–cell fusion is important for efficient cell-to-cell transmission and, therefore, the spreading infection of SARS-CoV-2.

Another interesting finding in this work is that although ACE2 enhances cell-to-cell transmission of SARS-CoV-2 and SARS-CoV, it is not absolutely required. This observation is supported further by data from H520 cells and human PBMCs, which express a minimal level of ACE2 if any, yet exhibited obvious cell-to-cell transmission of lentiviral pseudotyped virus (Fig. 4). Moreover, we obtained similar results by using authentic SARS-CoV-2 in H520 cells where clear cell-to-cell transmission was observed (Fig. 4). In all cases, cell-free infection of SARS-CoV-2 was not detected in H520 cells and PBMCs, further supporting these conclusions. The molecular mechanism underlying cell-to-cell transmission of SARS-CoV-2, including the possible roles of cellular cofactors and virological synapses, shall be investigated in future studies.

A surprising result to emerge from our studies was that, despite the critical role of cell–cell contact and plasma membrane–mediated fusion, endosomal entry pathways were also involved in cell-to-cell transmission of SARS-CoV-2 and SARS-CoV (Fig. 5). This is evidenced by the inhibitory effect of drugs that specifically target the endosomal entry pathway of these viruses, including the CatL inhibitor III, which blocks cleavage of the viral glycoprotein, as well as BafA1, which neutralizes endosomal pH. These results altogether are reminiscent of previous studies from HIV and EBOV, where the endocytosis and/or protease cleavage processed are required for cell-to-cell transmission of these enveloped viruses (26, 30, 71, 78, 79). Interestingly, we find that these inhibitors appear to be less potent for decreasing cell-to-cell transmission as compared with cell-free infection, including for authentic SARS-CoV-2 (Fig. 5 and *SI Appendix*, Fig. S4), and moreover, their effects on SARS-CoV-2 are weaker than their effects on SARS-CoV. We also noticed that the cell-free infection of SARS-CoV-2 in Vero-ACE2-TMPRRS2 cells was still sensitive to treatment by endosomal inhibitors, similar to a recent report (80); we reason that this could be associated with the levels of TMPPRS2 expression on the plasma membrane of target cells used for infection, as well as viral stocks that contain furin-defective mutants generated from Vero cells. These observations collectively suggest a less dominant role for the endosomal entry pathway in cell-to-cell transmission of SARS-CoV-2. High-resolution live microscopic imaging would be useful to dissect the exact role of endosomal vs. plasma membrane entry pathways in the cell-to-cell transmission of SARS-CoV-2.

In this work, we also tested the effect of remdesivir (RDV), an FDA-approved drug, on cell-to-cell infection of SARS-CoV-2. Previously, RDV has been shown to efficiently inhibit the replication of SARS-CoV-2 in Vero (half-maximal inhibitory concentration [IC_50_] =11.41 µM) and Calu-3 cells (IC_50_ = 1.1 µM) (81). Interestingly, we found only a modest effect of RDV on the cell-to-cell spread of authentic SARS-CoV-2, i.e., ∼50% inhibition from Vero-ACE2-TMPRSS2 to Calu-3 cells, even at a concentration of 100 µM (*SI Appendix*, Fig. S4 *D* and *E*). These results may not be so surprising, given the relatively shorter cococulturing time as well as the fact that RDV acts to block only the viral RNA-dependent RNA polymerase (RdRp) of newly transmitted virus in target cells, rather than the existing virus that was transferred from donor to target cells. Hence, the limited effect of RDV for cell-to-cell spread of SARS-CoV-2 supports the contention that authentic SARS-CoV-2 spreads efficiently through cell–cell contact.

Cell-to-cell transmission is considered to be an effective means by which viruses evade host immunity, especially antibody-mediated responses. We compared the sensitivity of cell-to-cell transmission vs. cell-free infection of SARS-CoV-2 to treatments by neutralizing monoclonal antibodies and COVID-19 convalescent plasma—both of which have been approved by the FDA for emergency use. We found that while cell-free infection of SARS-CoV-2 was almost completely blocked by these treatments, cell-to-cell transmission of SARS-CoV-2 was, to a large extent, refractory (Figs. 6 and 7). While not statistically significant, some of the COVID-19 sera (two out of five) even enhanced cell-to-cell transmission of SARS-CoV-2 (Fig. 6*D*), although the underlying mechanisms are currently not known. Interestingly, despite significant increases in cell-free infectivity, the South Africa variant B.1.351, the UK variant B.1.1.7, as well as the D614G variant, exhibited similar efficiencies of cell-to-cell transmission compared with the WT (Fig. 7). Moreover, although B.1.351 is more resistant to vaccinee sera in cell-free infection, consistent with some recent reports (67, 68), B.1.1.7 seems more resistant to the vaccinee sera for the cell-to-cell transmission route (Fig. 7). This may explain why B.1.1.7 has a longer duration of acute infection than other variants (82). The mechanism underlying these observations is currently unclear, but may have implications for understanding the rapid spread of VOCs in the human population as well as their increased pathogenesis. The cell-free route is directly linked to the ability of viruses to infect target cells and result in spreading among humans through person-to-person contact. In contrast, cell-to-cell transmission has dominant roles in viral pathogenesis and disease progression (24). Thus, our results on the resistance of B.1.1.7 and B.1.351 to vaccinee sera–mediated inhibition of cell-to-cell transmission and cell-free infection may provide molecular and virological underpinnings for the prolonged viral replication and rapid spread of these two variants in the world population (67, 68, 83, 84).

## Materials and Methods

Cell culture, virus, constructs, antibodies, and reagents, cell–cell fusion, plaque assay, flow cytometry, Western blotting, neutralization assay, and statistical analysis are described in *SI Appendix*, *SI Materials and Methods*.

### Cell-to-Cell Transmission.

In the lentiviral vector system, the expression of anti-sense reporter gene Gluc is interrupted by an intron oriented in the sense direction of the HIV-1 genome so that Gluc production will only occur in infected target cells and not virus producer cells (46). By coculturing the virus producer and target cells, cell-to-cell transmission was determined by measuring the Gluc activity of the cocultured media between donor cells (such as 293T) producing lentiviral pseudotypes and target cells (such as 293T/ACE2). Specifically, 293T cells were seeded in 6-well plates and transfected with 1.4 µg NL4.3-inGluc and 0.7 µg of plasmids encoding SARS-CoV or SARS-CoV-2 spike. The next day, transfected 293T donor cells were digested with phosphate-buffered saline (PBS)/5 mM ethylenediaminetetraacetic acid (EDTA) and thoroughly washed with PBS to remove EDTA, followed by coculturing with target cells (293T/ACE2, Caco-2, Calu-3, NCI-H520, or PBMCs) at a 1:1 ratio in 24-well plates for ∼24 to 72 h. Inhibitors or sera were added as needed. Supernatants were collected and measured for the Gluc activity.

For examining the cell-to-cell spread of authentic SARS-CoV-2 WT and VOCs, we infected the donor Vero-ACE2 cells with an MOI of 0.2 (WT) or 0.02 (VOCs) for 20 h and cocultured them with the same number of Vero-mTomato-Red cells for an additional 6 h, in the presence or absence of vaccinee sera. For the other purposes, Vero-ACE2-TMPRSS2-mTomato cells served as donor cells, which were infected with WT SARS-CoV-2 (USA-WA1/2020, MOI = 0.01) for 18 h, followed by being cocultured with Vero-ACE2-TMPRSS2, NCI-H520, or Calu-3 cells under specific conditions (see legends). Cells were then fixed with 3.7% formaldehyde for 1 h, followed by washing three times with PBS before being taken out of the BSL3 laboratory. The fixed cells were incubated with anti–SARS-CoV-2 nucleocapsid and anti-mouse-FITC, and subjected to flow cytometry analysis.

### Cell-Free Infection.

Cell-free infection was performed along with cell-to-cell transmission in this work. Briefly, an equal number of transfected donor cells were seeded in new 24-well plates and maintained for the same period of time as in cell-to-cell transmission (normally 48 to 72 h). The total volumes of supernatants were collected and used to infect target cells, which were seeded with the presence of the same amount of untransfected 293T cells; this would ensure that the total numbers of cells and density used for cell-to-cell and cell-free infection assays were comparable. For the Transwell setting, the transfected donor cells were seeded onto the insert while target cells, which again were mixed with the same amount of untransfected 293T cells, were on the bottom; this would avoid the contact between donor and target cells yet the virus can spread through the filter. Supernatants were collected at the same time points as cell-to-cell transmission and measured for Gluc activity.

## Supplementary Material

Supplementary File

## Data Availability

All study data has been deposited into the National Cancer Institute SeroNet website (https://www.immport.org/shared/study/SDY1890). The data will go live in January 2022 and it will be available upon request until then.
