# Supplementary material for: SARS-CoV-2 spreads through cell-to-cell transmission
Source: Proc Natl Acad Sci U S A. 2021 Dec 22;119(1):e2111400119. doi: 10.1073/pnas.2111400119 (PMC8740724; doi:10.1073/pnas.2111400119)
Supplement: Supplementary File [file pnas.2111400119.sapp.pdf]

Supplementary Information for

SARS-CoV-2 Spreads through Cell-to-Cell Transmission

Cong Zeng[1,2], John P. Evans[1,2,3], Tiffany King[4,5], Yi-Min Zheng[1,2], Eugene M. Oltz[6], Sean P. J. Whelan[7], Linda J. Saif [8, 9,10], Mark E. Peeples[4,5,10], and Shan-Lu Liu[1,2,6, 10 *]

* To whom correspondence should be addressed: Dr. Shan-Lu Liu

The Ohio State University, Columbus, OH 43210, USA,

Tel: (614) 292-8690; Email: liu.6244@osu.edu

This PDF file includes:

Supplementary text

Figures S1 to S5

SI References

Supplementary Materials and Methods

Cell culture. 293T (ATCC CRL-11268, RRID: CVCL_1926), Vero-E6 (ATCC CRL-1586, RRID: CVCL_0574) and Vero-ACE2 (Vero-E6 expressing high endogenous ACE2, BEI, NR-53726) cells were grown in Dulbecco's modified Eagle's medium (DMEM) supplemented with 1% penicillin/streptomycin and 10% (vol/vol) fetal bovine serum (Thermo Fisher Scientific). Caco-2 (ATCC HTB-37, RRID: CVCL_0025) cells were grown in Dulbecco's modified Eagle's medium (DMEM) supplemented with 1% penicillin/streptomycin and 20% (vol/vol) FBS. Calu-3 cells (ATCC HTB-55, RRID: CVCL_0609) were grown in Eagle's Minimum Essential Medium (EMEM) supplemented with 1% penicillin/streptomycin and 10% (vol/vol) FBS. De-identified human peripheral blood mononuclear cells (PBMCs) were gifts of Eric O. Freed (National Cancer Institute, Frederick, Maryland, USA) and maintained in Roswell Park Memorial Institute (RPMI) 1640 Medium containing 10% (vol/vol) FBS. NCI-H520 (ATCC HTB-182, RRID: CVCL_1566) cells were grown in RPMI 1640 Medium supplemented with 1% penicillin/streptomycin and 10% (vol/vol) fetal bovine serum. The 293T/ACE2 cell line was obtained from BEI (NR-52511). Vero-ACE2 cells stably expressing TMPRSS2 were generated by transduction of Vero-ACE2 cells with a lentiviral vector expressing human TMPRSS2, followed by blasticidin S HCl selection (7.5 μg/mL) for 7 days. Vero-E6 and Vero-ACE2-TMPRSS2 cells stably expressing red tomato were generated by transduction with a lentiviral vector expressing the tomato gene, followed by hygromycin B selection (200 μg/mL) for 6 days. All cell lines utilized were maintained at 37°C, 5% CO2.

Virus. rVSV-GFP-SARS-CoV and rVSV-GFP-SARS-CoV-2 (obtained from Sean Whelan's lab at the Washington University School of Medicine in St. Louis, Missouri, USA) were amplified in Vero-E6 cells and maintained under a humidified atmosphere of 5% CO_2 at 34°C in Dulbecco's modified Eagle's medium (DMEM) supplemented with 10% FBS. The spike sequence in the original stock and each passage was confirmed by DNA sequencing. Authentic SARS-CoV-2 WT (USA-WA1/2020, NR-52281; kindly prepared by Jacob Yount of The Ohio State University, Columbus, Ohio, USA), D614G (B.1.5, NR-53944), B.1.1.7 (alpha, 501Y.V1, NR-54000) and B.1.351 (beta, 501Y.V2, NR-54009) were all obtained from BEI.

Constructs, antibodies and reagents. HIV-1 NL4.3-inGluc was a gift of Marc Johnson at the University of Missouri (Columbia, Missouri, USA). Plasmids pcDNA3.1-SARS-CoV-S-C9 and pcDNA3.1-SARS-CoV2-S-C9 encoding the full-length spike were obtained from Fang Li at the University of Minnesota (St. Paul, Minnesota, USA). A construct for ACE2 transient expression, pHAGE2-ACE2, was obtained from BEI resources (NR-52512). A lentiviral vector encoding red tomato was from Marc Johnson (University of Missouri, Columbia, USA). The codon-optimized D614G, B.1.351 and B.1.1.7 SARS-CoV-2 S constructs were synthesized by GenScript and subsequently cloned into a pcDNA3.1 vector by restriction enzyme cloning with Kpn I and BamH I. Primary antibodies used for western blotting and flow cytometry were anti-coronavirus spike (Sino Biological, 40150-T62; now replaced by *40591-T62*), anti-SARS-CoV-2 Nucleocapsid (Sino Biological, 40143-MM08), anti-hACE2 (R&D, AF933) and anti-β-actin (Sigma, A1978). Secondary antibodies used for western blotting included anti-Mouse IgG-Peroxidase (Sigma, A5278), anti-Rabbit IgG-Peroxidase (Sigma,

A9169) and anti-Goat IgG-Peroxidase (Sigma, A8919). Secondary antibodies used for flow cytometry included anti-Rabbit IgG–FITC (Sigma, F9887), anti-Mouse IgG-FITC (Sigma, F0257), anti-Goat IgG-FITC (Sigma, F7367). The monoclonal Ab 2B04 was a gift of Ali Ellebedy (Washington University in St. Louis).

Inhibitors in this study included Methyl cellulose (Sigma, M0512), Cathepsin L Inhibitor III (Sigma, 219427), CA-074 Me (Sigma, 205531), EST/E-64D (Sigma, 330005), Bafilomycin A1 (Sigma, B1793), Leupeptin (Sigma, L2884), and Remdesivir (Selleckchem, GS-5734). EK1 peptide was synthesized by Alpha Diagnostic International (San Antonio, Texas).

Human serum samples. De-identified patient serum samples were collected from hospitalized COVID-19 patients under an approved IRB protocol #2020H0228 as described (1). De-identified vaccinee serum samples were collected from health care workers with consent following 3-4 weeks of the second dose of Moderna and Pfizer SARS-CoV-2 mRNA vaccination under the approved IRB protocols #2020H0228.

Cell-cell fusion. For fluorescence-based cell-cell fusion, 293T cells were transfected with plasmid encoding GFP and spikes. Following 24 hrs transfection, donor 293T cells were cocultured with target cells. Micrographs of cocultured cells were taken after 2~24 hrs coculture. For quantification of cell-cell fusion, we co-transfected donor 293T cells with a plasmid encoding a tetracycline-controlled transcription factor (tTA, also referred to Tet-off), along with plasmids encoding SARS-CoV or SARS-CoV-2 spike; target 293FT-mCAT-Gluc cells (stably expressing tetracycline-responsive element (TRE)-driven secreted *Gaussia* luciferase (Gluc)) were transfected with a plasmid expressing ACE2. Following 24 hrs post-transfection, donor 293T cells and target 293FT cells were cocultured at a 1:1 ratio; upon cell-cell fusion, the Gluc protein was expressed and secreted into the culture medium, which was detected by measuring the luciferase activity at 24 and 48 hr, respectively.

Plaque assay. The replication-competent rVSV-GFP-SARS-CoV and rVSV-GFP-SARS-CoV-2 viruses were used to infect confluent Vero-E6 cells (MOI=0.01) for 1 h at 37°C. The uninfected virus was then removed from cells and replaced with 1% methylcellulose in DMEM/5% FBS and incubated for 72 hr at 37°C. Cells were fixed with 3.7% paraformaldehyde in PBS and stained with 1% crystal violet (Sigma, C0775) in 10% ethanol for visualization of plaques.

Flow cytometry. For analysis of spike and ACE2 expression on the cell surface, transfected 293T cells were washed with PBS, detached with PBS/5mM EDTA for 10 min, washed twice with cold PBS/2% FBS, and incubated with anti-coronavirus Spike/Nucleocapsid or anti-hACE2 antibody for 1 hr. After three washes with cold PBS/2% FBS, cells were incubated with FITC-conjugated anti-rabbit IgG/anti-mouse IgG or anti-goat IgG (1:200) secondary antibodies for 1 hr. Cells were washed three times with cold PBS /2% FBS and fixed with 3.7% formaldehyde for 10 min and analyzed by flow cytometry. For analysis of rVSV-GFP-SARS-CoV and rVSV-GFP-SARS-CoV-2

infection, infected Vero E6 cells were washed with PBS and digested with 0.05% trypsin, followed by fixation with 3.7% formaldehyde for 10 min and analyzed by flow cytometry.

Western blotting. Western blotting was performed as previously described (1, 2). In brief, HEK293T cells were collected and lysed in RIPA buffer (50 mM Tris, pH 7.5, 150 mM NaCl, 1 mM EDTA, 1% Nonidet P-40, 0.1% SDS, protease inhibitor cocktail) for 40 min on ice, followed by centrifugation for 10 min, 12,000 x g at 4°C, Cell lysate then boiled at 100 ℃ for 10 min with 1XSDS loading buffer containing 2-Mercaptoethanol. Samples were run on 10% SDS-PAGE gels, transferred to PVDF membranes, and probed with primary antibodies and secondary antibodies, analyzed by Amersham Imager 600 (Thermofisher).

Neutralization assays. Cell-free virus neutralization assays were performed by incubating free virus with serial diluted Moderna and Pfizer vaccinee sera, followed by infecting 293T/ACE2 target cells and measuring the luciferase activity (1) at 48 and 72 hr. Cell-to-cell virus neutralization assays were performed by incubating serial diluted sera with viral producer cells (transfected 293T) and target cells (293T/ACE2) in the coculture system, and supernatants were collected at 48 and 72 hr to measure the luciferase activity. In both cases, NT_{50} was defined as the sera dilution fold at which the relative light units were reduced by 50% compared with the control wells (no sera); the NT_{50} values were calculated using nonlinear regression in GraphPad Prism.

Statistical Analysis. Data were analyzed as mean with Standard Error of Mean (SEM). All experiments were performed at least three independent replications, and the number of biological replicates for each data set is given by "n" and is provided in the respective figure legend. Statistical analyses were performed using GraphPad Prism 5.0 as follows: One-way Analysis of Variance (ANOVA) with Bonferroni's post-tests was used to compute statistical significance between multiple groups for multiple comparison or t-test was used for two groups for single comparison. A p value of less than 0.05 was considered significant and indicated by an asterisk (*, $p<0.05$).

Supplemental Figures

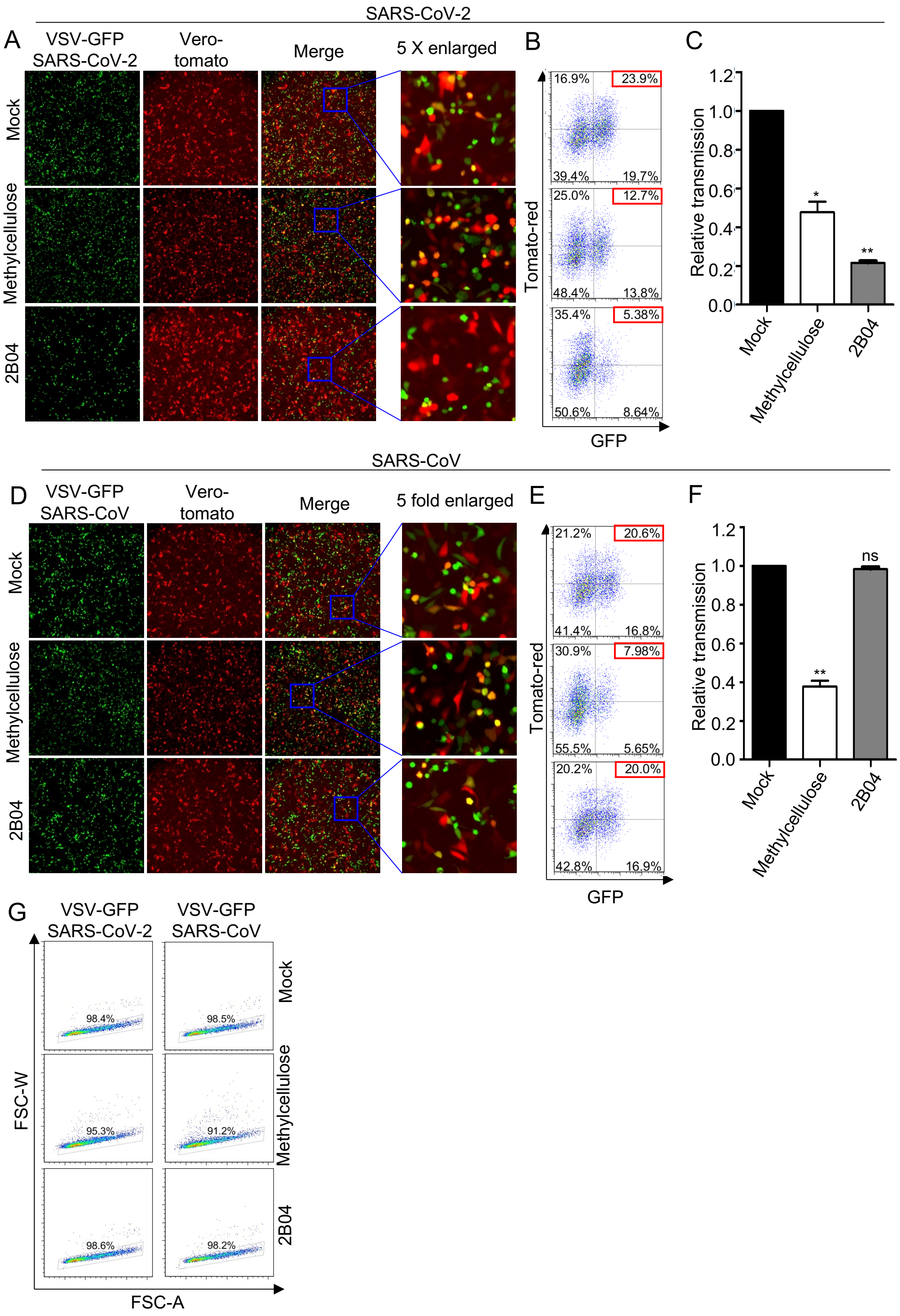

Figure S1. Effects of methylcellulose and monoclonal antibody 2B04 on rVSV-GFP transmission in Vero-E6 cells. Vero-E6 cells were infected with appropriate MOIs of either VSV-GFP-SARS-CoV or VSV-GFP-SARS-CoV-2. After 16 h post-infection, the infected Vero-E6 cells were cocultured with Vero-mTomato-Red cells at 1:1 ratio, in the presence or absence of 2 µg/mL 2B04 or 1% methylcellulose. Micrographs of cocultured cells were taken after 18 h coculture (**A and D**), with dual fluorescence positive cells indicated by arrows. The GFP signals in Tomato-positive cells were analyzed by flow cytometry (**B and E**, Q2), indicative of virus transmission from Vero-E6 to Vero-mTomato-Red cells. Results from 3 independent experiment (n=3) were summarized and plotted as relative infection rates by setting the values of mock infection control to 1.0 (**C and F**). (**G**) FSC-A vs FSC-W analysis of cell population for Figs. S1B and 1E.

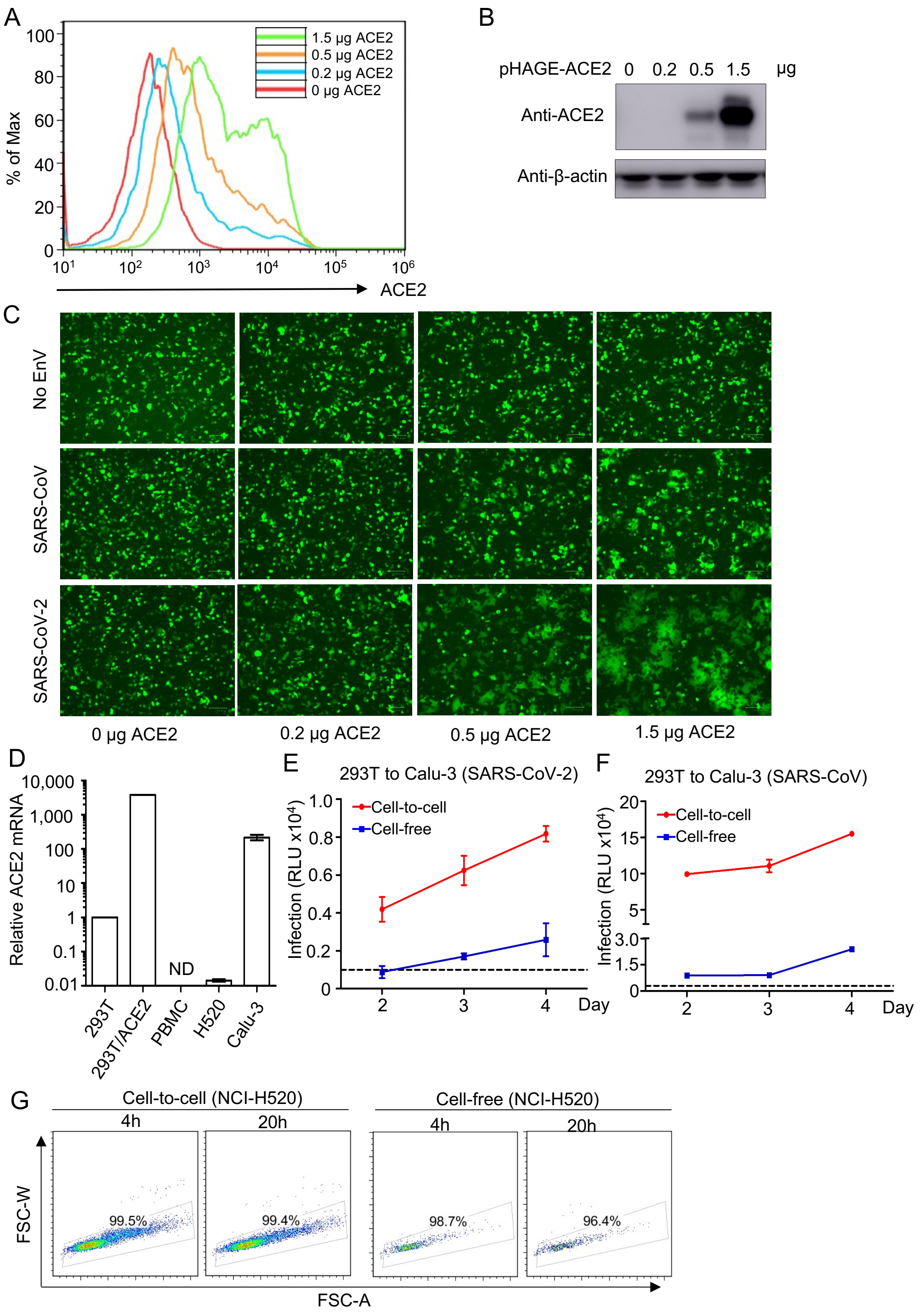

Figure S2. Role of ACE2 in cell-to-cell transmission. (**A and B**) The expression level of ACE2 in target cells was analyzed by flow cytometry (**A**) and western blotting (**B**) using a specific antibody

against ACE2; results were one representative of three independent experiments. (**C**) Representative images of cell-cell fusion induced by SARS-CoV-2 and SARS-CoV spike at indicated doses of ACE2. (**D**) The expression level of ACE2 in different cell lines and human PBMCs. qPCR was performed to quantify the ACE2 mRNA expression and relative expression was plotted by setting the value of 293T cells to 1.0. ND: not detected. (**E and F**) Cell-to-cell transmission in Calu-3 cells. Experiments were performed as described in Figures 1 and 4, except that Calu-3 cells were used as target cells, which were cocultured with viral producer 293T cells (n=3). (**G**) FSC-W vs FSC-A analysis for figure 4G.

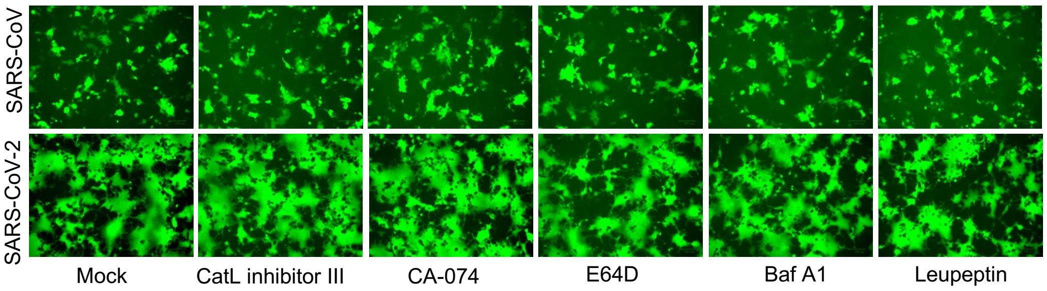

Figure S3. Effect of endosomal entry inhibitors on cell-cell fusion induced by SARS-CoV-2 spike. Experiments were carried out as described in Figures 3 and 5, with indicated inhibitors included in the cell coculture: 5 μM Cat L inhibitor III, 5 μM CA-074, 30 μM E-64D, 50 nM BafA1, and 50 μM leupeptin.

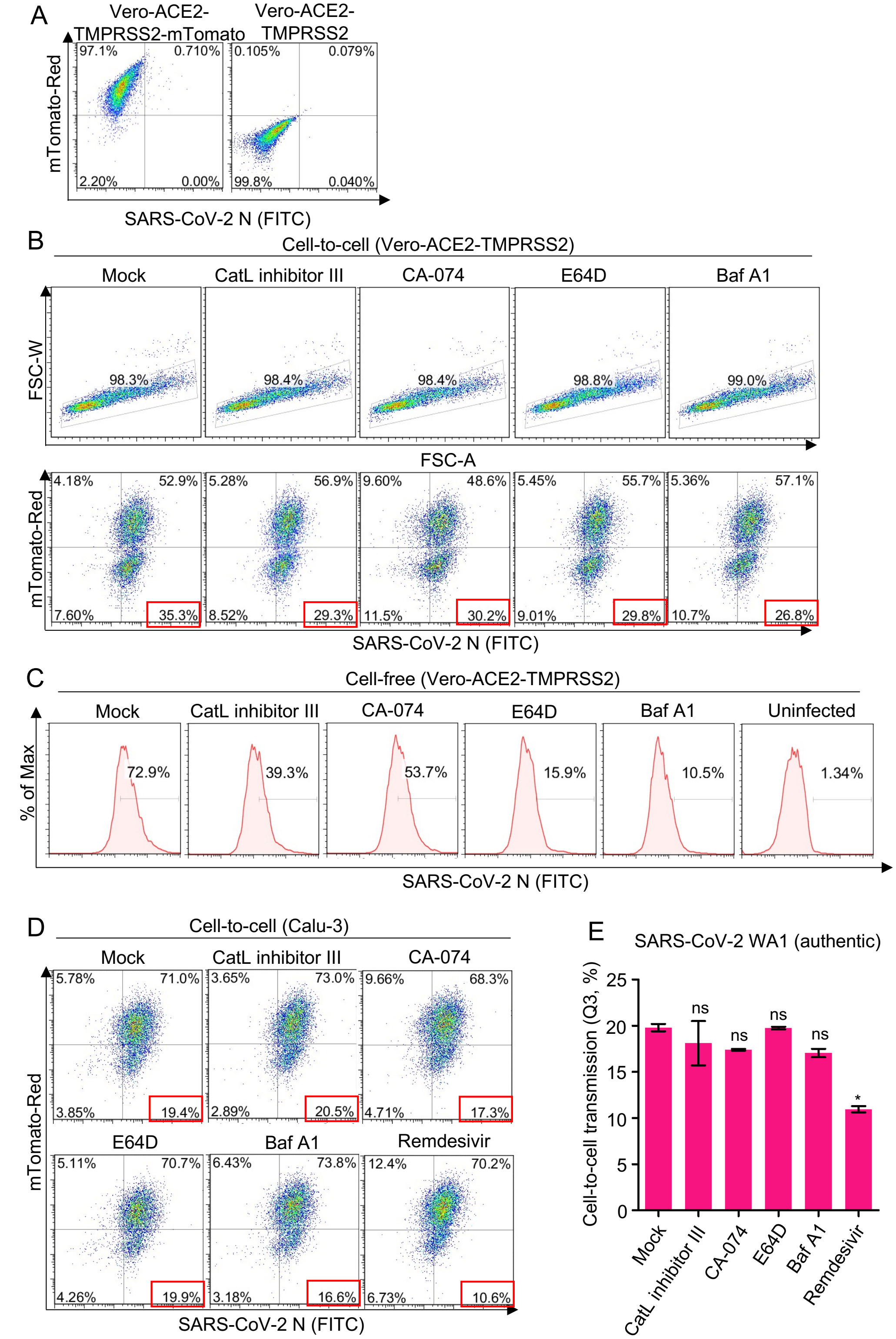

Figure S4. Effect of endosomal entry inhibitors on cell-to-cell transmission of authentic SARS-CoV-2 in cells expressing TMPRSS2. (**A-C**) Vero-ACE2-TMPRSS2-mTomato (Red) cells were infected with MOI=0.01 of authentic SARS-CoV-2 WT (USA-WA1/2020) for 18 hour, followed

by co-culturing them with Vero-ACE2-TMPRSS2 cells in presence of 5 μM CatL inhibitor **III**, 5 μM CA-074, 25 μM E64D or 50 nM Baf A1 for another 4 hours. In parallel, same amounts of Vero-ACE2-TMPRSS2 target cells were infected with SARS-CoV-2 in presence of these inhibitors for 6 hours to measure cell-free infection. Cells were fixed and stained with anti-SARS-CoV-2 N protein for flow cytometric analysis. (**A**) Flow cytometric gating controls using uninfected donor Vero-ACE2-TMPRSS2-mTomato (Red) and target Vero-ACE2-TMPRSS2 cells. (**B**) Representative flow cytometric analyses of cell-to-cell transmission. FSC-A vs FSC-W analysis was used to ensure single cells. (**C**) Representative flow cytometric analysis of cell-free infection. (**D and E**) Cell-to-cell transmission of authentic SARS-CoV-2 from Vero-ACE2-TMPRSS2-mTomato cells to Calu-3 in presence of 5 μM CatL inhibitor **III**, 5 μM CA-074, 25 μM E64D, 50 mM Baf A1 or 100 μM Remdesivir. The same experimental procedure was applied as described in (B) except that Calu-3 cells were used as target cells, which were mixed with donor cells at a ratio of 1:4. Note that the cell-free infection in Calu-3 cells was too low to be presented because of limited cell numbers seeded for infection as well as a relatively shorter period of infection.

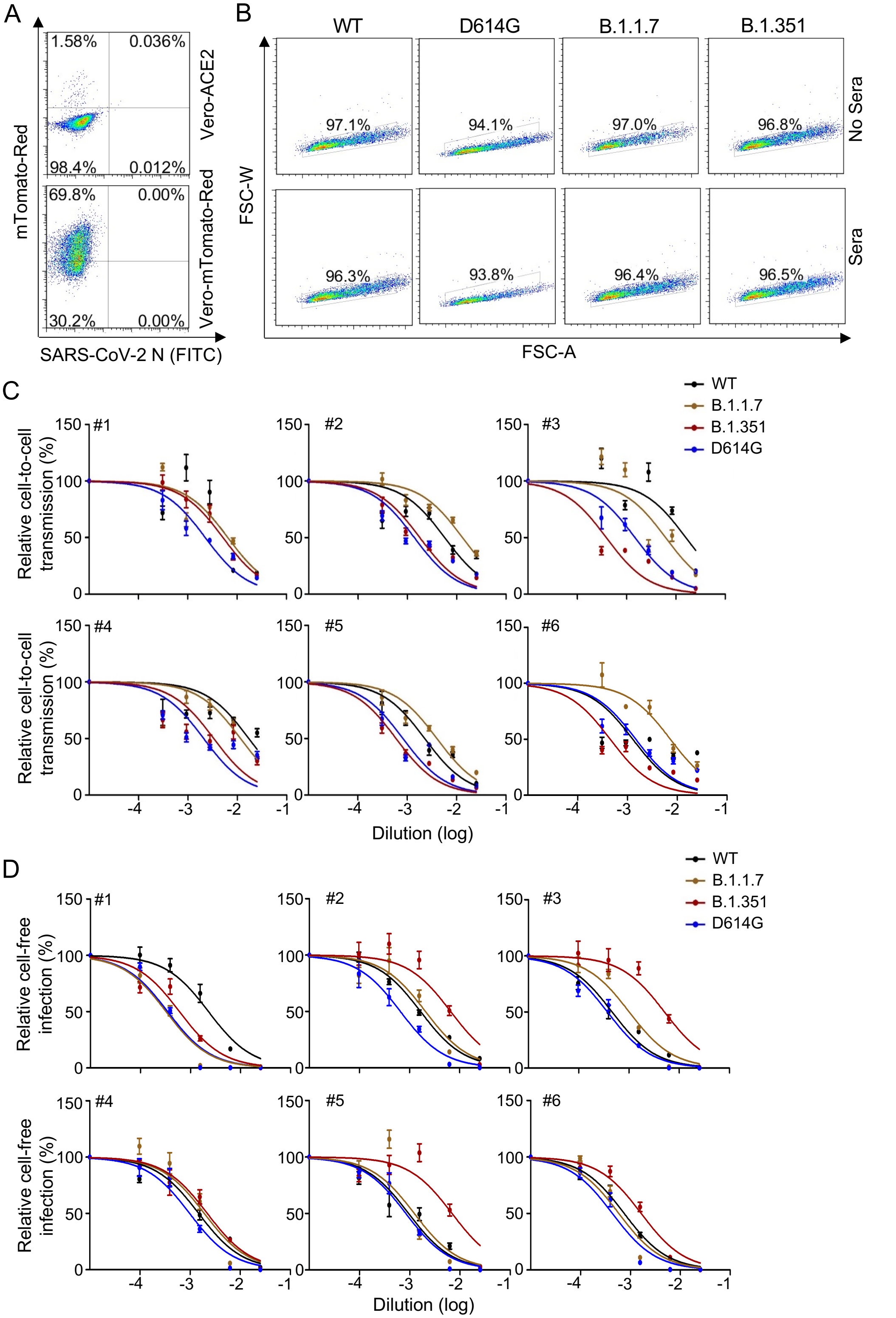

Figure S5. Neutralization curves of vaccinee sera against the cell-to-cell and cell-free infection of VOCs B1.1.7 and B.1.351 relative to D614G and WT. (**A**) Flow cytometric gating

control in analysis of data presented in Figure 7A using uninfected Vero-ACE2 and Vero-mTomato-Red cells. (**B**) FSC-A vs FSC-W analysis of cell populations for Figure 7A (**C and D**). Six vaccinee sera samples, 3 from Moderna and 3 from Pfizer, were chosen for the neutralization assay in the context of cell-to-cell transmission or cell-free infection. The *y* axis indicates the relative viral infectivity by setting the viral infectivity without serum to 100%; the *x* axis indicates dilution fold of serum samples (n=6).

Supplementary References

1. C. Zeng *et al.*, Neutralizing antibody against SARS-CoV-2 spike in COVID-19 patients, health care workers, and convalescent plasma donors. *JCI Insight* **5** (2020).
2. M. Li *et al.*, TIM-mediated inhibition of HIV-1 release is antagonized by Nef but potentiated by SERINC proteins. *Proc Natl Acad Sci U S A* **116**, 5705-5714 (2019).
